# Knowledge of Interpreter Rights and Medication Delays in Diabetes Care

**DOI:** 10.1007/s10903-026-01905-z

**Published:** 2026-03-27

**Authors:** Miguel A. Linares, Estelle M. Everett, Jorge A. Rodriguez, Alejandra Casillas, Arleen Brown

**Affiliations:** 1 University of California, Los Angeles, USA; 2 Brigham and Women’s Hospital, Boston, USA

**Keywords:** Interpreter rights, Non-English language preference, Immigrants, Diabetes

## Abstract

To examine the association between knowledge of interpreter rights and diabetes care quality and health care utilization among adults with non-English language preference (NELP) in California. We conducted a cross-sectional analysis using pooled data from the 2015–2024 California Health Interview Survey (CHIS). The study included adults with NELP, self-reported diabetes, and a usual source of care. The primary exposure was self-reported knowledge of rights to free medical interpretation. Outcomes included self-reported receipt of hemoglobin A1c, eye, and foot exams; delays or forgone medical care or prescription medications; and measures of health care utilization. Survey weighted Poisson regression models were used to estimate adjusted risk ratios (RRs) and 95% confidence intervals (CIs), controlling for sociodemographic, health, and utilization-related covariates. Among 2,379 adults with diabetes and NELP (representing an average annual weighted population of 508,871), 76.6% reported knowledge of interpreter rights. In unadjusted analyses, lack of knowledge was associated with a higher likelihood of delayed prescribed medications (RR 1.76, 95% CI 1.20–2.58). This association persisted after adjustment (aRR 1.81, 95% CI 1.24–2.63). No significant associations were observed for other diabetes care metrics or health care utilization outcomes. Associations did not differ by diabetes type. Among adults with diabetes and NELP, lack of knowledge of interpreter rights was associated with delays in obtaining prescribed medications. Interpreter rights awareness may represent a modifiable factor to improve engagement in communication-dependent aspects of diabetes care and advance language equity in health care delivery.

**Supplementary Information:**

The online version contains supplementary material available at 10.1007/s10903-026-01905-z.

Language preference represents a critical but often overlooked determinant of diabetes care quality [1]. Compared to those with English language preference, adults with non-English language preference (NELP) face disproportionate obstacles to effective communication in clinical settings, which may limit their ability to understand disease management plans, navigate health systems, access new technologies and adhere to recommended care [1–4]. These communication barriers contribute to poorer glycemic control and lower rates of preventive screenings [3, 5, 6]. Professional medical interpreters can improve communication, patient engagement, and some clinical outcomes [7]. Their use is protected under federal language access mandates, including Title VI of the Civil Rights Act of 1964 and Sect. 1557 of the Affordable Care Act [8]. However, interpreter services are not consistently offered, and their utilization may be influenced by patient knowledge of their rights to an interpreter [9, 10].

In the context of diabetes, guideline-based care involves a combination of regular clinical monitoring and preventive assessments that depend on clear communication between patient and clinician, such as hemoglobin A1c (HbA1c) testing, and foot and eye examinations [11]. These services are essential for the early detection and management of complications that, if missed, can progress to blindness, kidney failure, neuropathy, lower-extremity amputation, and premature mortality [11]. The effectiveness of such preventive services partially relies on patients’ ability to understand and act on clinical guidance, which in turn hinges on language-concordant communication. When language discordance is unaddressed and patients lack knowledge about their right to request a qualified interpreter at no cost, their access to health care may be compromised [10]. While differences in diabetes-related outcomes between individuals with NELP and ELP have been previously documented [1, 3, 4], little is known about whether knowledge of interpreter rights, a potentially modifiable factor, facilitates self-advocacy and improves uptake of recommended diabetes services. This study examines the association between self-reported knowledge of the right to medical interpretation and indicators of diabetes care quality and health care utilization among adults with NELP in California.

## Research Design and Methods

### Study Design and Participants

We conducted a cross-sectional analysis using pooled data from the 2015–2024 California Health Interview Survey (CHIS), an annual, population-based survey designed to provide representative estimates of the noninstitutionalized population of California. CHIS employs a multistage sampling design using random-digit dialing of landline and cellular telephones and online questionnaires, and is administered in 6 non-English languages: Spanish, Cantonese, Mandarin, Korean, Tagalog, and Vietnamese.

Our study population included adult respondents (≥ 18 years) who met three criteria: (1) self-reported NELP, defined as speaking English “not well” or “not at all” on the CHIS English proficiency item; (2) reported having a usual source of medical care, identified through the question, *“Is there a place that you usually go to when you are sick or need advice about your health?”*; and (3) self-reported a diagnosis of diabetes.

### Exposure

Participants were included if they responded to the CHIS question on interpreter rights: *“In California*,* you have the right to get help from an interpreter for free during your medical visits. Did you know this before today?”* The primary exposure was self-reported knowledge of this right, categorized as “yes” or “no.”

### Outcomes

We evaluated eight self-reported diabetes-related care and utilization metrics over the past 12 months. Metrics included having at least one hemoglobin HbA1c test, an eye examination, and a foot examination, as well as delaying or forgoing necessary prescribed medications or medical care. Health care utilization metrics included having a personal doctor, any physician visit and an emergency department (ED) visit during the same 12-month period. All outcomes except the HbA1c test and foot examination were available across survey years 2015–2024; the HbA1c test was assessed for the years 2015, 2016, and 2019–2024, and the foot examination was assessed in the 2015–2018 cycles.

### Covariates

Covariates were selected a priori and included sociodemographic variables (age, sex, race and ethnicity, marital status, educational attainment, household income as a percentage of the federal poverty level, employment status, rural or urban geography and number of years in the U.S.), insurance status, self-rated health status (excellent, very good, good, fair, poor) and survey year [12]. Language was excluded due to collinearity with race and ethnicity. For all adjusted risk ratio models, except the outcome of any physician visit, the number of physician visits in the past 12 months was included as a covariate to account for differences in health care utilization that may influence both knowledge of interpreter rights and receipt of diabetes-related services.

### Statistical Analysis

Analyses incorporated CHIS-provided survey weights to account for the complex sampling design and to generate annual population-level estimates. Participant characteristics were summarized by knowledge of interpreter rights using weighted proportions, and group differences were assessed with Rao-Scott chi-square tests. We estimated adjusted risk ratios (RRs) and 95% confidence intervals (CIs) using survey-weighted Poisson regression models [13] for the association between knowledge of interpreter rights and each outcome, controlling for all covariates. Effect modification by diabetes type was evaluated using interaction terms. For missing data, we performed a complete case analysis as missingness was less than 5% for all outcomes and covariates. Statistical significance was defined as a two-sided *P* value < 0.05. All analyses were conducted using Stata version 17.0. The use of publicly available, deidentified data qualified this study for exemption from institutional review board oversight.

## Results

### Participant Characteristics

The study included 2,379 adults with NELP in California, a usual source of care, and self-reported diabetes, representing an average *annual* weighted population of 508,871 individuals across the 10-year study period. Of these, 389,846 (76.6%) reported knowledge of their rights to free medical interpretation and 119,025 (23.4%) reported no such knowledge (Table 1).

**Table 1:** Weighted annual baseline characteristics of participants by knowledge of rights to interpretation: 2015–2024 California health interview survey

Characteristic	Knowledge of Rights to interpretation		No Knowledge of Rights to Interpretation		
	Unweighted *n* = 1,862 weighted *N* = 389,846		Unweighted *n* = 517 weighted *N* = 119,025		*P*
	Unweighted n	Weighted %	Unweighted n	Weighted %	
*Sex*					0.15
Male	807	42.8%	237	49.2%	
Female	1,055	57.2%	280	50.8%	
*Age*					0.61
18–39	76	5.5%	16	3.2%	
40–49	182	13.7%	57	12.0%	
50–64	731	39.8%	195	45.1%	
≥ 65	873	41.0%	249	39.7%	
*Insurance*					0.04
Uninsured	140	8.0%	62	13.9%	
Insured	1,722	92.0%	455	86.1%	
*Education*					0.64
Less than HS	1,073	77.4%	318	74.2%	
HS	344	13.3%	97	16.2%	
Some College	194	4.6%	39	5.8%	
College	251	4.7%	63	3.8%	
*Years in US*					0.47
< 10	102	6.1%	34	6.2%	
10–14	96	5.6%	29	7.6%	
> 15	1,664	88.4%	454	86.2%	
*Geography*					0.05
Urban	1,827	99.3%	512	99.8%	
Rural	35	0.7%	5	0.2%	
*Poverty Level*					0.29
< 100	759	41.4%	211	42.9%	
100–199	699	37.5%	178	31.4%	
200–299	199	10.1%	56	11.8%	
≥ 300	205	11.1%	72	14.0%	
*Race and Ethnicity*					0.01
Hispanic	1,330	79.2%	344	71.9%	
Asian	501	18.6%	160	26.6%	
Other^†^	31	2.2%	13	1.4%	
*Employment*					0.83
Employed	1,255	34.2%	341	35.2%	
Unemployed	607	65.8%	176	64.8%	
*Language*					0.04
Spanish	1,022	61.1%	264	49.1%	
Chinese	149	6.1%	47	6.9%	
Vietnamese	78	3.6%	34	7.8%	
Korean	141	2.9%	38	4.1%	
English and another	369	20.8%	106	26.5%	
≥ 1 non-English	103	5.4%	28	5.6%	

^†^Includes individuals self-identifying as non-Hispanic African American or White.

**Table 2:** Risk Ratios (RR) for lack of knowledge of interpreter rights and diabetes care metrics and health care utilization among adults with diabetes

	RR (95% CI)	*P*	RR (95% CI)	*P*
	Unadjusted		Adjusted	
HbA1c test ≥ 1^†^	1.02 (0.97, 1.07)	0.50	1.00 (0.95, 1.05)	0.98
Eye examination	0.98 (0.90, 1.06)	0.56	0.98 (0.91, 1.06)	0.61
Foot examination^††^	0.84 (0.68, 1.03)	0.09	0.86 (0.70, 1.06)	0.15
Delayed or forgone prescribed medications	1.76 (1.20, 2.58)	0.004	1.81 (1.24, 2.63)	0.002
Delayed or forgone medical care	1.27 (0.82, 1.97)	0.27	1.26 (0.77, 2.05)	0.35
Has a personal physician	0.96 (0.88, 1.05)	0.40	0.99 (0.91, 1.07)	0.75
Any physician visit	0.98 (0.93, 1.03)	0.39	0.98 (0.93, 1.03)	0.43
Emergency Dept. visit	0.98 (0.74, 1.29)	0.88	0.96 (0.71, 1.30)	0.79

The reference for all comparisons is having knowledge of interpreter rights.

Metrics reflect diabetes monitoring (A1c, eye, foot exams), health care utilization (physician and Emergency Dept. visits), and barriers to needed prescriptions or medical care within the past 12 months.

^†^Analysis limited to survey years in which the question was administered: 2015-2016, 2019-2024.

^††^Analysis limited to survey years in which the question was administered: 2015-2018.

Compared with those with knowledge of their interpreter rights, individuals without knowledge were more likely to be uninsured (13.9% vs. 8%, *P* = .04). The distribution of sex, age, education level, years in the United States, urban vs. rural residence, poverty level, and employment status did not differ significantly between groups. By race and ethnicity, a higher proportion of participants without knowledge identified as Asian (26.6% vs. 18.6%) and a lower proportion as Hispanic (71.9% vs. 79.2%, *P* = .01). Spanish was the predominant language in both groups, though more common among those with knowledge of their rights (61.1% vs. 49.1%).

### Diabetes Care Outcomes

In unadjusted analyses, lack of knowledge of interpreter rights was significantly associated with a higher likelihood of delays in obtaining prescribed medications (RR = 1.76, 95% CI 1.20–2.58, *P* = .004, Table 2). There was no significant unadjusted association with receipt of HbA1c testing, eye and foot examinations, delays in medical care, having a personal physician, any physician visit or ED visit in the past 12 months.

In the adjusted models, lack of knowledge remained significantly associated with a higher likelihood in delays obtaining prescribed medications (aRR = 1.81, 95% CI 1.24–2.63, *P* = .002). Adjusted associations with HbA1c testing, eye and foot examinations, delays in medical care, having a personal physician, any physician visit or ED visits in the past 12 months were not statistically significant.

### Effect Modification by Diabetes Type

Among respondents in survey years when diabetes type was assessed (2015–2022), 87% (weighted *N* = 345,743) reported type 2 diabetes and 13% (weighted *N* = 51,526) reported type 1 diabetes. Effect modification by diabetes type was evaluated by including an interaction term between knowledge of interpreter rights and diabetes type in the survey-weighted Poisson models for each outcome. The interaction term was not statistically significant for any outcome (all interaction *P* values > 0.15, Supplemental Table 1), indicating no evidence that associations differed by diabetes type.

## Discussion

This study is among the first to examine how knowledge of rights to medical interpretation relates to the quality of diabetes care among adults with NELP, a population that faces well-documented barriers to communication, health literacy, and access within the U.S. health system [14]. In a large, representative sample of adults with diabetes, we found that those with no knowledge of their interpreter rights had a higher likelihood of delays in obtaining prescribed medications.

Knowledge of interpreter rights may serve as a proxy for a specific dimension of health literacy, namely, the capacity to understand and act upon legal entitlements to language services in health care. While conventional definitions of health literacy emphasize skills such as reading and numeracy [15], this form of knowledge reflects more targeted competencies like navigating health systems and engaging in self-advocacy [16, 17]. In our sample, formal educational attainment did not differ significantly between those with and without interpreter rights knowledge, suggesting that this awareness is not simply a byproduct of traditional educational attainment [18] but may stem from other exposures, such as prior use of interpreters, targeted outreach from health institutions, or community-based information sharing.

In this diabetes-focused study, lack of interpreter rights knowledge was associated with a higher likelihood of delays in obtaining prescribed medications, though not with other diabetes care and health care utilization measures evaluated. Importantly, this association persisted even after adjustment for household income as a percentage of the federal poverty level, suggesting that the observed relationship is not fully explained by economic disadvantage alone [19]. In the context of diabetes, delays in filling or picking up prescribed medications are associated with poorer glycemic control and higher rates of long-term complications including retinopathy, nephropathy, neuropathy, cardiovascular events, and even mortality [20, 21]. Taken together, the findings suggest that the association may be most pronounced for processes that require active communication and coordination outside the clinic between the patient and their clinician or care team and has been previously documented in previous research. Notably, in a large cohort study of adults with diabetes, poorer patient-clinician communication was independently associated with inadequate refills of cardiometabolic medications, particularly for oral hypoglycemics [22]. Additionally, prior work among Latinos with NELP has shown that interpreter use significantly increases the likelihood of receiving important information when a new medication is prescribed, including explanations of its purpose, possible side effects, and dosing instructions, which may help prevent treatment delays and improve adherence [23]. Overall, these findings indicate that communication-related barriers to timely medication access for adults with diabetes may be at least partially mitigated by interpreter rights knowledge.

In diabetes care, HbA1c, retinal and foot preventive screenings are vital components of preventative management [11]. Importantly, no differences were found in the adjusted and unadjusted models. The absence of an HbA1c association may reflect this test’s routine incorporation into routine visits. Prior work from has shown that Spanish-preferring Latinos had higher rates of HbA1c testing than both non-Latino Whites and English-preferring Latinos, even after adjusting for covariates. Yet Spanish-preferring Latinos also had disproportionately higher rates of uncontrolled HbA1c levels [5]. This suggests that higher testing rates do not necessarily translate into better glycemic control and that persistent disparities may be driven by broader barriers to effective disease management, including linguistic and cultural discordance [6]. 

Consistent with earlier research, our analysis of health care access measures revealed no significant differences in health care access. No differences were found in ED use between those with and without knowledge of interpreter rights [10]. This finding may reflect the ED’s role as a safety net for individuals with NELP, providing a point of access for acute care needs regardless of other barriers [24]. The absence of differences in having a personal physician and any physician in the past 12 months further suggests that simply having a designated source of care may not translate into regular engagement. Interpreter rights awareness could nonetheless play a role in shaping how patients navigate and utilize available primary care services.

While the responsibility for ensuring language access rests with the health care system, awareness of rights to medical interpretation remains critical for self-advocacy, particularly when institutional safeguards fail or are inconsistently implemented. Although a prior single-year California survey found no association between legal awareness and interpreter use [25], our findings suggest that a lack of awareness of interpreter rights is negatively associated with some health services. This is relevant to evidence that individuals with NELP may internalize perceptions of “undeservingness” when navigating exclusionary health systems, eroding their any sense of entitlement to accommodations [26]. Such internalized barriers may limit the impact of access-oriented policies unless paired with efforts to strengthen patients’ knowledge, confidence and agency to assert their rights. From this perspective, interpreter rights knowledge reflects not only informational access but may also impact the capacity and willingness to act on it. This makes interventions that build both awareness and self-efficacy especially important for ensuring that having a usual source of care translates into receipt of all recommended diabetes services.

This study has several implications. On the one hand, interpreter rights awareness may be a modifiable factor in adherence to recommended diabetes care. Interventions could include multilingual educational campaigns, integration of rights information into chronic disease self-management programs, and visible prompts in clinic settings. Screening for interpreter rights awareness as part of social determinants of health assessments could help identify patients at risk for underutilizing preventive services. Simultaneously, system-level strategies are needed to ensure that interpreter services are normalized and seamlessly integrated into routine care, such as embedding automatic prompts for interpreter offers within electronic health record workflows, monitoring interpreter use as a quality metric and ensuring institutional accountability for compliance with language access standards [27]. Since communication barriers intersect with other determinants of health, such as immigration status, socioeconomic disadvantage, and health literacy, addressing this knowledge should be part of a broader, multilevel approach that combines patient-level education with structural reforms to promote language equity.

Limitations of this study include its cross-sectional design, which precludes causal inference, and reliance on self-reported measures, which may be subject to recall bias. CHIS data are representative of California but may not generalize to other states with different interpreter service availability or language access policies. Additionally, the dataset lacked information on certain potentially relevant factors, such as diabetes duration, and clinical indicators of disease control, which could influence service utilization. Despite these limitations, this study provides new evidence that awareness of interpreter rights is associated with timely access to health services among NELP populations.

In conclusion, among California adults with diabetes and NELP, lack of knowledge of rights to medical interpretation was associated with a higher likelihood of prescribed medication delays. Knowledge of interpretation rights among individuals with NELP may be a modifiable factor to improve engagement in communication-dependent diabetes care, and targeted outreach could help ensure language access policies translate into equitable care delivery.

## Supplementary Information

Below is the link to the electronic supplementary material.

Supplementary Material 1

## Data Availability

No datasets were generated or analysed during the current study.

## References

[1] Holman H, Müller F, Bhangu N, Kottutt J, Alshaarawy O. Impact of limited English proficiency on the control of diabetes and associated cardiovascular risk factors. The National Health and Nutrition Examination Survey, 2003–2018. Prev Med. 2023;167:107394. 10.1016/j.ypmed.2022.107394. 36563970 10.1016/j.ypmed.2022.107394

[2] Rodriguez JA, et al. Language Disparities in Continuous Glucose Monitoring for Type 2 Diabetes. JAMA Netw Open. 2025;8:e2516523. 10.1001/jamanetworkopen.2025.16523. 40526387 10.1001/jamanetworkopen.2025.16523 PMC12175012

[3] Parker MM, Fernández A, Moffet HH, Grant RW, Torreblanca A, Karter AJ. Association of Patient-Physician Language Concordance and Glycemic Control for Limited–English Proficiency Latinos With Type 2 Diabetes. JAMA Intern Med. 2017;177(3):380. 10.1001/jamainternmed.2016.8648. 28114680 10.1001/jamainternmed.2016.8648 PMC5339062

[4] Njeru JW, et al Diabetes Mellitus Management Among Patients with Limited English Proficiency: A Systematic Review and Meta-Analysis. J Gen Intern Med. 2018;33(4):524–32. . 10.1007/s11606-017-4237-1. 29256089 10.1007/s11606-017-4237-1 PMC5880756

[5] Aceves B, et al. Disparities in HbA1c testing between aging US Latino and non-Latino white primary care patients. Prev Med Rep. 2022;26:101739. 10.1016/j.pmedr.2022.101739. 35295668 10.1016/j.pmedr.2022.101739 PMC8918837

[6] Fernandez A, et al. Language Barriers, Physician-Patient Language Concordance, and Glycemic Control Among Insured Latinos with Diabetes: The Diabetes Study of Northern California (DISTANCE). J Gen Intern Med. 2011;26(2):170–6. 10.1007/s11606-010-1507-6. 20878497 10.1007/s11606-010-1507-6 PMC3019330

[7] Hacker K, et al. Exploring the Impact of Language Services on Utilization and Clinical Outcomes for Diabetics. PLoS ONE. 2012;7:e38507. 10.1371/journal.pone.0038507. 22675571 10.1371/journal.pone.0038507 PMC3366945

[8] Pope TM. Patients with Limited English Proficiency: Legal Mandates for Language Assistance Services. Am J Bioeth. 2024;24(11):78–80. 10.1080/15265161.2024.2399833. 39401723 10.1080/15265161.2024.2399833

[9] Garrison E, Bey A, Jimenez E, Li A, Nettles A. Results of a multidisciplinary workshop to reduce bias and improve health equity for patients with limited english proficiency [ID: 1375774]. Obstet Gynecol. 2023;141(5S):94S. 10.1097/01.AOG.0000931148.27353.d4

[10] Linares M, Lipsitz S, Shaykevich S, Samal L, Rodriguez JA. Knowledge of Interpreter Rights and Health Care Access. JAMA Netw Open. 2025;8(7):e2519244. 10.1001/jamanetworkopen.2025.19244. 40622716 10.1001/jamanetworkopen.2025.19244 PMC12235493

[11] Solomon SD, et al. Diabetic Retinopathy: A Position Statement by the American Diabetes Association. Diabetes Care. 2017;40(3):412–8. 10.2337/dc16-2641. 28223445 10.2337/dc16-2641 PMC5402875

[12] Linares M, Lipsitz S, Shaykevich S, Samal L, Rodriguez JA. Knowledge of Medical Interpretation Rights Among Individuals With Non-English Language Preference: A Cross-Sectional Study. Med Care. 2025;63(3):249–55. 10.1097/MLR.0000000000002109. 39739585 10.1097/MLR.0000000000002109

[13] Cummings P. The Relative Merits of Risk Ratios and Odds Ratios. Arch Pediatr Adolesc Med. 2009;163(5):438. 10.1001/archpediatrics.2009.31. 19414690 10.1001/archpediatrics.2009.31

[14] Sentell T, Braun KL. Low Health Literacy, Limited English Proficiency, and Health Status in Asians, Latinos, and Other Racial/Ethnic Groups in California. J Health Commun. 2012;17(sup3):82–99. 10.1080/10810730.2012.712621. 23030563 10.1080/10810730.2012.712621 PMC3552496

[15] Martin LT, et al. Patient Activation and Advocacy: Which Literacy Skills Matter Most? J Health Commun. 2011;16(3):177–90. . 10.1080/10810730.2011.604705 21951251 10.1080/10810730.2011.604705 PMC3187917

[16] Schmidt EK, Faieta J, Tanner K. Scoping Review of Self-Advocacy Education Interventions to Improve Care. OTJR Occup Ther J Res. 2020;40(1):50–6. 10.1177/1539449219860583. 10.1177/1539449219860583 31342850

[17] Dumez V, L’Espérance A. Beyond experiential knowledge: a classification of patient knowledge. Soc Theory Health. 2024;22(3):173–86. 10.1057/s41285-024-00208-3.

[18] Dessalegn K, Girma B, Oumer KE, Hunie M, Belete KG. Patients’ awareness of their rights, associated factors and its practice by health professionals from a patient perspective among elective surgical patients at Tikur Anbessa Specialized Hospital, Addis Ababa, Ethiopia: a cross-sectional study, 2021. BMJ Open. 2022;12(11):e060218. 10.1136/bmjopen-2021-060218. 36428024 10.1136/bmjopen-2021-060218 PMC9703307

[19] Lyles CR, et al. Financial Strain and Medication Adherence among Diabetes Patients in an Integrated Health Care Delivery System: The Diabetes Study of Northern California (DISTANCE). Health Serv Res. 2016;51(2):610–24. 10.1111/1475-6773.12346. 26256117 10.1111/1475-6773.12346 PMC4799896

[20] Paul SK, Klein K, Thorsted BL, Wolden ML, Khunti K. Delay in treatment intensification increases the risks of cardiovascular events in patients with type 2 diabetes. Cardiovasc Diabetol. 2015;14(1):100. 10.1186/s12933-015-0260-x. 26249018 10.1186/s12933-015-0260-x PMC4528846

[21] Kaewbut P, Kosachunhanun N, Phrommintikul A, Chinwong D, Hall JJ, Chinwong S. Time to Treatment Intensification to Reduce Diabetes-Related Complications: A Post Hoc Study. Healthcare. 2022;10(9):1673. 10.3390/healthcare10091673. 36141285 10.3390/healthcare10091673 PMC9498838

[22] Ratanawongsa N, et al. Communication and Medication Refill Adherence: The Diabetes Study of Northern California. JAMA Intern Med. 2013;173(3):210. 10.1001/jamainternmed.2013.1216. 23277199 10.1001/jamainternmed.2013.1216 PMC3609434

[23] Moreno G, Tarn DM, Morales LS. Impact of Interpreters on the Receipt of New Prescription Medication Information Among Spanish-Speaking Latinos. Med Care. 2009;47(12):1201–8. 10.1097/MLR.0b013e3181adcc1b. 19786911 10.1097/MLR.0b013e3181adcc1b PMC2951837

[24] Njeru JW, et al. Emergency department and inpatient health care utilization among patients who require interpreter services. BMC Health Serv Res. 2015;15(1):214. 10.1186/s12913-015-0874-4. 26022227 10.1186/s12913-015-0874-4 PMC4448538

[25] Grubbs V, Chen AH, Bindman AB, Vittinghoff E, Fernandez A. Effect of awareness of language law on language access in the health care setting. J Gen Intern Med. 2006;21(7):683–8. 10.1111/j.1525-1497.2006.00492.x. 16808766 10.1111/j.1525-1497.2006.00492.x PMC1924696

[26] Kline N. When deservingness policies converge: US immigration enforcement, health reform and patient dumping. Anthropol Med. 2019;26(3):280–95. 10.1080/13648470.2018.1507101. 31550907 10.1080/13648470.2018.1507101

[27] Gutman CK, et al. Strategies to Increase Professional Interpreting in Clinical Settings: A Systematic Review. JAMA Netw Open. 2025;8:e2521492. 10.1001/jamanetworkopen.2025.21492. 40674051 10.1001/jamanetworkopen.2025.21492 PMC12272290

