# Supplementary material for: Knowledge of Interpreter Rights and Medication Delays in Diabetes Care
Source: J Immigr Minor Health. 2026 Mar 27;28(3):696–701. doi: 10.1007/s10903-026-01905-z (PMC13222275; doi:10.1007/s10903-026-01905-z)
Supplement: Supplementary file 1 — Supplementary Material 1 [file 10903_2026_1905_MOESM1_ESM.docx]

**Supplemental Table 1. Effect Modification by Diabetes Type in Risk Ratios (RR) for Lack of Knowledge of Interpreter Rights and Diabetes Care Metrics and Health Care Utilization Among Adults with Diabetes, CHIS 2015–2022ᵃ,ᵇ**

|  | Adjusted Interaction Risk Ratios, (95% CI) | *P* |
| --- | --- | --- |
| HbA1c test ≥ 1† | .93 (.73, 1.18) | .52 |
| Eye examination | .91 (.72, 1.15) | .43 |
| Foot examination †† | 1.35 (.63, 2.93) | .44 |
| Delayed or forgone prescribed medications | .30 (.06, 1.57) | .15 |
| Delayed or forgone medical care | .66 (.11, 3.90) | .64 |
| Has a personal physician | .87 (.66, 1.15) | .32 |
| Any physician visit | 1.02 (.66, 1.57) | .93 |
| Emergency Dept. visit | .86 (.29, 2.52) | .78 |
| ^a^ The reference for all comparisons is having knowledge of interpreter rights among adults with type 1 diabetes. Diabetes type was assessed in survey years 2015-2022. | | |
| ^b^ Metrics reflect diabetes monitoring (A1c, eye, foot exams), health care utilization (physician and Emergency Dept. visits), and barriers to needed prescriptions or medical care *within the past 12 months.* | | |
| † Analysis limited to survey years in which the question was administered: 2015-2016, 2019-2024.  †† Analysis limited to survey years in which the question was administered: 2015-2018. | | |
